# Evaluation of Maternal Health Service Indicators in Urban Slum of Bangladesh

**DOI:** 10.1371/journal.pone.0162825

**Published:** 2016-10-12

**Authors:** Saira Parveen Jolly, Mahfuzar Rahman, Kaosar Afsana, Fakir Md Yunus, Ahmed M. R. Chowdhury

**Affiliations:** 1 Research and Evaluation Division, BRAC, Mohakhali, Dhaka 1212, Bangladesh; 2 Health, Nutrition and Population Program, BRAC, Mohakhali, Dhaka 1212, Bangladesh; 3 Corporate Office, BRAC, Mohakhali, Dhaka 1212, Bangladesh; Aalto University Helsinki Institute for Information Technology, FINLAND

## Abstract

**Background:**

A continuous influx of poor people to urban slums poses a challenge to Bangladesh’s health system as it has failed to tackle maternal morbidity and mortality. BRAC is the largest non-governmental organisation in Bangladesh. BRAC has been working to reduce maternal, neonatal and under-five children morbidity and mortality of slum dwellers in cities. BRAC has been doing this work for a decade through a programme called MANOSHI. This programme provides door-to-door services to its beneficiaries through community health workers (CHWs) and normal delivery service through its delivery and maternity centres. BRAC started the ‘MANOSHI’ programme in Narayanganj City Corporation during 2011 to address maternal, neonatal and child health problems facing slum dwellers. We investigated the existing maternal health-service indicators in the slums of Narayanganj City Corporation and compared the findings with a non-intervention area.

**Methods:**

This cross-sectional study was conducted during 2012, in 47 slums of Narayanganj City Corporation as intervention and 10 slums of Narsingdi Sadar Municipality as comparison area. A total of 1206 married women, aged 15–49 years, with a pregnancy outcome in the previous year were included for interview. Data on socio-demographic characteristics, reproductive and maternal health-care practices like use of contraceptive methods, antenatal care (ANC), delivery care, postnatal care (PNC) were collected through a structured questionnaire. The chi-square test, Student *t* test, Mann Whitney U-test, factor analysis and log-binominal test were performed by using STATA statistical software for analysing data.

**Results:**

The activities of BRAC CHWs significantly improved four or more ANC (47% vs. 21%; p<0.000) and PNC (48% vs. 39%; p<0.01) coverage in the intervention slums compared to comparison slums. Still, about half of the deliveries in both areas were attended at home by unskilled birth attendants, of which a very few received PNC within 48 hours after delivery. The poorest and illiterate women received fewer maternal health services from medically trained providers (MTPs). The poorest had a lower likelihood of receiving services from MTPs during delivery complications.

**Conclusion:**

The MANOSHI programme service coverage for delivery care and PNC-checkup for women who prefer home delivery needs to be improved. For sustainable improvement of maternal health outcomes in urban slums, the programme needs to facilitate access to services for poor and illiterate women.

## Introduction

Millennium Development Goal-5 (MDG- 5) emphasised the reduction of maternal mortality by three-fourths between 1990 and 2015 [1]. Still maternal health remains one of the main global health challenges worldwide. In 2015 alone, 303,000 maternal deaths were witnessed [2]. During 2015 Sub-Saharan Africa and Southeast Asia contributed 59% of total maternal deaths across the world and Bangladesh ranked 10th in the top ten countries with the most maternal mortality (5500 uncertainty interval 3900 to 8800) [1]. Post 2015, the agenda on Sustainable Development Goals (SDGs) includes absolute reduction in mortality as a marker of progress [1]. Therefore, it is time to focus efforts in low and middle-income countries helping them to progress towards achieving the SDGs. Most maternal deaths occur during pregnancy, and or after 42 days of delivery [1]. Earlier studies have suggested that access to quality healthcare in pregnancy, childbirth and the postnatal period may yield multiple returns on investment by reducing maternal and neonatal deaths as well as improving child development outcomes [3, 4]. Thus, maximum utilisation of maternal health care services within the existing health system, eradicating poverty, improving literacy and empowerment of women will help in expediting the progress towards attaining SDGs to reduce preventable maternal deaths [5].

Although, Bangladesh remained one of the top ten countries in the world for high maternal deaths, early 2015 progress towards MDG-5 was on track and maternal mortality rate was reduced from 322 to 194 per 100,000 live births in a decade (2001–2010) [6]. During that time, the annual reduction rate was 5–6%, which was higher than expected to achieve MDG-5 by 2015 [6]. Therefore, it is high time to identify the reasons behind successes in maternal health services in Bangladesh those that were effective for reduction of maternal morbidity and mortality. Earlier studies showed that countrywide combined efforts of stakeholders including Government, Non-Government organisations (NGOs), and international agencies were able to improve women’s education, access to basic emergency obstetric care (EmOC), comprehensive EmOC and promotion of maternal health services at the tertiary, secondary and community level through medically trained providers (MTPs) and community health workers (CHWs) [6–9]. These endeavors resulted in improved maternal healthcare services namely use of modern family planning methods, antenatal care (ANC) checkups by MTPs, skilled assisted delivery, postnatal care (PNC) checkup and referrals to facilities with obstetric care during complications [10, 11].

In order to reduce the equity gap in maternal health services, marginalised women need more access to health services. The demographic health survey in Bangladesh showed that improvements in maternal health services is better in urban areas, compared to rural areas (11). Despite this progress in maternal health, the urban poor in Bangladesh exhibit dire pregnancy outcomes and a greater maternal mortality rate compared to their urban counterparts [12, 13]. A recent urban health survey in Bangladesh reported clear differences in maternal health services amongst women of slum and non-slum areas, e.g. access to ANC with MTPs being 29% in the slums compared to 58% in non-slum areas, delivery at a facility (37% vs. 65%) and skilled assisted delivery (37% vs. 68%) [14]. This disparity is due to a lack of access to affordable health services for the slum population, their limited knowledge of available services and misconceptions around accessing healthcare facilities [15, 16]. In Bangladesh, rapid urbanisation has forced poor rural people to migrate to urban areas and settle in various slums. It is predicted that, half of Bangladesh’s population will live in cities by 2050 [14]. Although, private health facilities are proliferating rapidly in urban areas of Bangladesh, which are actually unaffordable for the slum poor people, in contrary public facilities are growing very slowly. Thus resulting in limited access to quality care for the poor [17]. Therefore, maternal healthcare issues are not being tackled for a significant proportion of the urban population. This hinders the achievement of the SDG.

### Overview of the BRAC implemented MANOSHI program

BRAC is the largest NGO in the Bangladesh and collaborated with Government of Bangladesh in order to improve the health of marginalised people. In 2007, BRAC established MANOSHI, a community-based Maternal, Neonatal, and Child Health (MNCH)–a care service package to reduce both maternal and child morbidity and mortality targeting slum-populations across city corporations in Bangladesh [15, 18–21]. It utilises female CHWs to promote family planning methods and provides door-to-door ANC and PNC checkups to women. They are paid very small remuneration for their assigned tasks. They are assigned to visit 200 households in a month and 15 households in a day. After identification of pregnancy of women, BRAC CHWs visit them at home to provide them monthly ANC checkups until delivery and after delivery; they visit to provide PNC-checkups for both mothers and neonates. They supply calcium tablets and iron-folic acid tablets during ANC checkups. The CHWs provide physical examinations, advice for biochemical tests and council pregnant women on maternal and neonatal healthcare practices during ANC checkups. They encourage women to have skilled assisted deliveries and supply delivery kits for safe delivery at home. They also identify maternal complications and refer them to facilities for appropriate treatment. Each slum is equipped with a delivery or a maternity centre for preventing unsafe home deliveries. In these facilities, the MANOSHI midwife and Urban Birth Attendant assist delivery cases. BRAC CHWs provide an ANC card to their beneficiaries to record services that they receive during the pregnancy and postnatal periods. The MANOSHI programme was scaled up in May 2011 in the Narayanganj City Corporation.

### Study objectives

The MANOSHI programme has been successful in reducing neonatal mortality, increased skilled assisted ANC checkup, and delivery, and reduced referral delays in targeted intervention areas [12, 18, 19, 21]. Despite its success, the programme requires improvement in its guidelines to meet the needs of women in the intervention area. The current study aimed to explore the status of maternal health service indicators among women living in the slums of Narayanganj City Corporation and compared the findings with a non-intervention area of similar setup, but also to provide information to improve the intervention if necessary to reach its full potential.

## Methods

### Study design and area

We conducted a community based cross-sectional comparative study in 2012 where we computed and compared the differences of maternal health service indicators between with MANOSHI provided health services and without MANOSHI services. MANOSHI had been implemented in all slums of Narayanganj City Corporation, which were considered an intervention area. The comparison area was selected from the slums of Narsingdi Sadar Municipality. In Narayanganj City Corporation, there were 94-slums and 35-health facilities including government, private and NGO clinics. On the contrary, in Narsingdi Sadar Municipality, there were only 10 slums and 31 health facilities. The literacy rate of Narayanganj City Corporation and Narsingdi Sadar Municipality was comparable (65% vs. 62%) and their degree of poverty was similar [22]. Before 2011, BRAC health programme had already implemented MANOSHI programme in all City Corporations of Bangladesh and Narayanganj was a newly declared city corporation. Therefore, we had to choose a comparison area that was adjacent to intervention area. We assumed that both areas might have similar health care practices and experienced a secular trend of MNCH or sudden change in health sector.

### Sample size

“Bangladesh Urban Health Survey” is a sample survey of urban slum and non-slum population conducted in 2006 suggested that 600 women from each slum area would require reaching at 80% statistical power with a 5% level of significance, 1.5 inter-cluster correlation, and 5% non-response rate [23]. This sample size was determined based on the proportion of four or more ANC checkups by MTPs among women who lived in urban slum areas.

### Sampling technique

**Fig 1:**
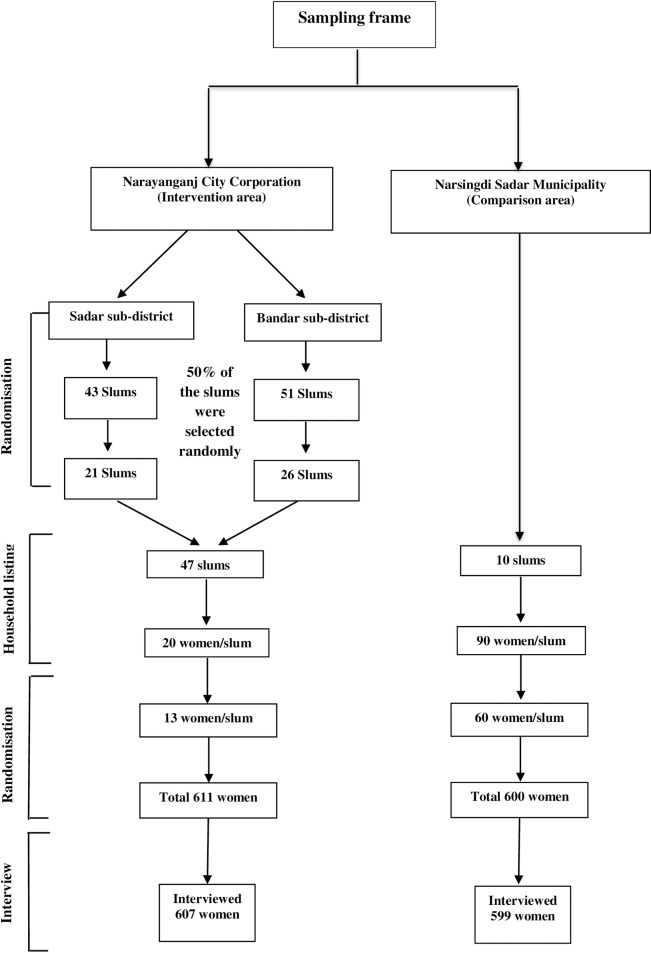
Sampling and randomization for selecting the respondents.

A two-stage cluster random sampling was used to select the respondents for this study. A computer generated random number was used to select 47 slums from the 94 slums of the intervention area. The scheme used to identify the relevant women is outlined in Fig 1. A house-to-house survey (household listing) was conducted ten days prior to the interview to identify eligible mothers (married women aged 15–49 years and having a pregnancy outcome during one-year preceding interview). The household listing was carried out until 20 eligible mothers were identified in each slum. We included five types of pregnancy outcomes in the household listing 1) infants aged under 12-months (live or deaths), 2) abortion, 3) menstrual regulation, 4) still birth, and 5) intrauterine death. An abortion was defined as the intentional interruption of a pregnancy by the application of external agent whether chemical or physical, or the ingestion of chemical agents with an intention other than to produce a live birth or to remove a dead fetus, regardless of the length of gestation [24]. In contrast, a menstrual regulation was defined as a form of vacuum aspiration, used to empty the uterus to pass the entire menses at once and it is administered by an MTP [25]. According to the definition of international classification of disease (ICD), stillbirth refers to a baby born with no signs of life at or after 28 weeks of gestation [2]. In addition, intrauterine death was defined as death prior to the complete expulsion or extraction from its mother of a product of human conception, irrespective of the duration of pregnancy and this is not an induced termination of pregnancy [25]. The intrauterine death is indicated by the fact that after such expulsion or extraction, the fetus does not breathe or show any other evidence of life, such as beating of the heart, pulsation of the umbilical cord, or definite movement of voluntary muscles. Heartbeats are to be distinguished from transient cardiac [25]. One potential challenge of researching in the slum was the frequent migration of its residents. In order to avoid difficulties arising from the migration of slum dwellers and to minimise the cost this study preferred not to include the whole slums in the sampling frame. However, to get 20 eligible mothers we had to enlist 300–400 households. Thirteen mothers were selected randomly from these 20 identified eligible mothers of each slum, and a total of 607 respondents were interviewed. Of these, 566 women had a living child under-one year of age, six had a child under one-year who had died within a year of the interview, and there were 13 abortions, 16 menstrual regulations, four stillbirths, and two intrauterine deaths. All the ten slums were included in this study. Ninety eligible women were identified from each of the Narsingdi Sadar Municipality sites and out of that number, 60 of them were selected randomly for interview (Fig 1). A total of 599 women were interviewed, and of these 519 had a living child aged under one-year, 12 women had a child who died before one-year. Besides, there were 48 abortions, 10 menstrual regulations, four stillbirths, and six intrauterine deaths.

### Data collection tools and quality control

A pre-structured questionnaire was used for this survey. The validated questionnaires of the Bangladesh Demographic Health Survey [26] and the MANOSHI midline survey 2009 [27] were considered during the development of the survey tool. The contents of the questionnaire were reviewed and modified to resemble the objective of the current survey. Further modification of the survey was carried out after pre-test by the field enumerators in neighboring slums.

Twenty-four female interviewers and four male field supervisors were recruited on the basis of their prior experience in the MNCH survey for in-person interviews. They received comprehensive training for nine days, followed by field practice and reporting feedback for two-days in order to cohere to the question and its format strictly, with the same degree of questioning on the objective measurement for both intervention and comparison areas. The mode of training consisted of lectures, mock interviews, and written exams. The interviewers were then divided into two groups, each headed by two field supervisors for the different intervention and comparison areas. Another interviewer and her supervisor crosschecked each data form. Two field operation officers and an investigation team were based at the study sites for monitoring the performance of the field staffs and for quality control. They spot checked and reinterviewed 10% of mothers within two days of the initial interview and randomly checked the data forms. If there was inconsistency or incompleteness within the collected data, respondents were re-interviewed in both intervention and comparison areas. Daily meetings with field staff and investigators were held at the local BRAC office to address any problems that arose in the field and share new experiences.

Several methods were used to reduce the non-response rate. First, efforts were made to convince the household heads of the importance of the interviews, a step that facilitated responses from the mothers. Second, working mothers were asked to choose a time for their interview. Lastly, in case of failure of obtaining information from a mother, another respondent was selected from the household survey list (even though she was not assigned randomly).

### Outcome variables

The outcome variables consisted of a proportion of current use of contraceptive, provision of ANC checkup, assistance at delivery, action against complications arising from delivery and PNC checkup either from MTPs, (which included doctor, nurse, Family Welfare Visitor (FWV) and midwife) or from other providers such as BRAC’s own CHWs (locally called *Shasthaya Kormi*), or trained providers (which included both MTPs and BRAC CHWs) or other NGO workers.

### Statistical analysis

A wealth index based on the ownership of household assets is widely recognised as a proxy for household economic status [28, 29]. In order to get wealth index, some variables such as, property, household assets, household construction materials, water, sanitation, and fuel supply were changed into dichotomous variables. Later, factor analysis was used to assign weighting values to indicator variables. The wealth quintile was constructed using the rank procedure. Similar analyses were performed to assess the quality of ANC. This analysis was conducted based on the receipt of: 1) four or more ANC check-ups from MTPs; 2) presence of several ANC examinations; 3) iron-folic acid tablets; 4) tetanus toxoid; and 5) advice on several issues. The quality of ANC was categorized into tertiles and the lowest, middle and highest tertile were defined as poor, moderate and better quality respectively. The skewed or non-normal continuous variables were tested using the Mann- Whitney U non-parametric test and data were presented as the median, range and a p-value. Analysis of the parametric continuous variables was performed using the Student’s *t*-test and results showed as Mean±SD with a p-value. All the categorical variables were analysed using the chi-square (χ^2^) test and the result expressed as percentage, a number, and p-value. The association between indicators and predictors was analysed using the log-binominal model and the data expressed as a risk ratio (RR) with 95% confidence interval (CI). The interpretation of odds ratio as risk ratio was not considered appropriate because odds ratio overstated risk ratio, as it was a cross-sectional study [30]. A database of dichotomous variables was constructed for this analysis. The value of ‘1’ was given for any woman receiving any of the maternal health service indicators; otherwise, the value ‘0’ was used. In the case of the predictor variables, the upper segment was given a value of ‘1’ otherwise ‘0’. This analysis was performed using STATA Version 12 (Chicago Inc.). Significance was taken at p<0.05.

### Ethical Issues

Research and Evaluation Division (RED), BRAC approved the proposal following existing rules. The eligible mothers were informed about the study during house-to-house surveys and informed verbal consent was obtained. Before interview, field enumerators comprehensively explained to each respondent about the nature of the programme, rationale of the study, questionnaire, and the risk and benefit of the study in front of a witness. Once they voluntarily agreed they were then asked to sign or thumbprint.

## Results

The socio-demographic details of the respondents of both areas are summarised in Table 1. The mean age of the women was similar in both areas. Their median years of schooling was 5.0, and 64.2% of mothers in both areas could read and write. The majority of the women in both areas were homemakers with only a few women involved in earning. Watching television was common in both areas whereas few of the women listened to radio or read newspapers. More than a quarter of the women in both areas were involved with micro-credit activities run by different NGOs. Their socio-economic status was comparable (p = 0.488).

**Table 1:** Socio-demographic characteristics of the respondents.

Study Variables		Intervention slum, n = 607		Comparison slum, n = 599		P-value
Age in years, % (n) *						
	≤19	16.97	(103)	18.03	(108)	0.094
	20–34	78.75	(478)	74.96	(449)	
	≥ 35	4.28	(26)	7.01	(42)	
Mean age, in years (Mean±SD)**		24.24	±5.01	24.57	±5.45	0.275
Marital status, % (n)*						
	Married	99.18	(602)	98.83	(592)	0.474
	Others ^a^	0.82	(5)	1.17	(7)	
Literacy						
	Can read and write, % (n)*	64.25	(390)	63.77	(382)	0.863
	Year of schooling, in year, [median (range)]***	5	(0–14)	5	(0–14)	0.523
Involved in earning, % (n)*		10.38	(63)	9.68	(58)	0.687
Main occupation, % (n)*						
	Housewife	91.60	(556)	94.82	(568)	0.001
	Others ^b^	8.4	(51)	5.18	(31)	
Exposure to media, % (n)						
	Watch television	89.95	(546)	87.15	(522)	0.126
	Listen to radio	1.81	(11)	0.83	(5)	0.138
	Read newspaper	6.75	(41)	8.35	(50)	0.295
	Involved with NGO	26.52	(161)	29.72	(178)	0.218
Socio-economic status, % (n)*						
	1^st^ quintile	18.78	(114)	20.37	(122)	0.488
	2^nd^ quintile	18.12	(110)	21.20	(127)	
	3^rd^ quintile	20.43	(124)	18.20	(109)	
	4^th^ quintile	20.59	(125)	20.53	(123)	
	5^th^ quintile	22.08	(134)	19.70	(118)	

* χ2 test

**Student’s *t* test

*** Mann Whitney U test

^a^ Divorced, separated and widowed

^b^ Day laborer, skilled laborer, garment laborer, craftswomen, service, small business, beggar, maid servant, tailor, tutor. Intervention slum: Slums of Narayanganj City Corporation; Comparison slum: Slums of Narsingdi Sadar Municipality

Early marriage was prevalent in both areas, with the average age of first marriage being 16.8 and 16.3 years in the intervention and comparison slums respectively (p <0.001; Table 2). The age of the first conception was 18 years in the intervention slums and 17.7 years in the comparison slums (p = 0.001). During the survey period, modern contraceptive prevalence rate was higher in intervention slums compared to comparison slums (64% vs. 50%; p <0.001). Fewer women in the intervention slums experienced one or more abortions compared to the comparison slums (13.5% vs. 19.2%; p = 0.005). Comparable proportion of women in both areas experienced one or more menstruation regulation, stillbirth, and intrauterine death. Importantly, a higher proportion of women in the comparison slums experienced one or more under-five child death compared to women in the intervention slums (5.4% vs. 11.7%; p <0.001; Table 2).

**Table 2:** Family planning and reproductive history of the respondents.

Study Variable		Intervention slum		Comparison slum		P-value
Number of respondents, N		607		599		
Age at first marriage, in year (Mean±SD)**		16.89	±2.70	16.34	±2.71	<0.001
Age at first conception, in year (Mean±SD)**		18.29	±2.83	17.75	±2.91	0.001
Couple currently using any FP method, % (n)*		66.39	(403)	55.09	(330)	<0.001
Number of married adolescent girls, N		22		38		
Married adolescent girls currently using modern FP method, % (n)*		68.18	(15)	39.47	(15)	0.032
Number of respondents, N		607		599		
Currently using modern FP method ^a^, % (n)*		63.80	(387)	49.90	(299)	<0.001
Proportion of each method, % (n)*						
	Pill	30.00	(182)	27.50	(165)	<0.001
	Injection	15.70	(95)	10.40	(62)	
	Condom	14.00	(85)	7.50	(45)	
	Intrauterine device	0.80	(5)	0.20	(1)	
	Implant	1.00	(6)	1.50	(9)	
	Female sterilization	1.80	(11)	2.30	(14)	
	Male sterilization	0.50	(3)	0.50	(3)	
Number of child ever born, median (range)***		2	(1–5)	2	(1–6)	0.005
Experienced one or more abortion, % (n)*		13.51	(82)	19.20	(115)	0.005
Total number of abortions, N		101		151		
Type of abortion, % (n)*						
	Spontaneous	92.10	(93)	82.10	(124)	0.025
	Induced	7.90	(8)	17.90	(27)	
Experienced one or more MR, % (n)*		7.91	(48)	6.01	(36)	0.196
Experienced one or more still birth, % (n)*		2.14	(13)	4.01	(24)	0.060
Experience one or more IUD, % (n)*		2.47	(15)	2.17	(13)	0.729
Experienced one or more child deaths, % (n)*		6.10	(37)	12.20	(73)	<0.001
Experienced one or more under 5 child deaths, % (n)*		5.40	(33)	11.70	(70)	<0.001
Number of children who died, N		43		97		
Age stratification of child death, % (n)*						
	0–7 days	27.90	(12)	37.10	(36)	0.081
	8–28 days	9.30	(4)	23.70	(23)	
	29–364 days	18.60	(8)	15.50	(15)	
	1–4 years	25.60	(11)	13.40	(13)	
	5 years and more	18.60	(8)	10.30	(10)	

* χ2 test

**Sudent’s *t* test

*** Mann Whitney U test

FP-family planning; IUD–intra uterine death; MR- Menstruation regulation. Intervention slum: Slums of Narayanganj City Corporation; Comparison slum: Slums of Narsingdi Sadar Municipality

The provision of ANC is summarized in Table 3. Although the majority of women in both areas received tetanus injections both before and during pregnancy, a significantly higher proportion of women in the intervention slums received iron and folic acid tablets during pregnancy (65.9% vs. 46.9%; p < 0.001). Similarly, a higher number of women in the intervention slums received at least a single ANC check-up from an MTP compared to their counterpart (79.7% vs. 56.8%; p <0.001). Only 9.4% of women in the intervention area and 0.8% women in the comparison area received ANC from BRAC CHWs (p <0.001). The number of women receiving four or more ANC check-ups from MTPs, BRAC CHWs and trained providers was also significantly higher in the intervention slums compared to those in the comparison slums (p <0.001). A quality ANC checkup comprises of the receipt of: 1) four or more ANC check-ups from MTPs; 2) presence physical, biochemical and ultrasound examinations; 3) iron-folic acid tablets; 4) tetanus toxoid; and 5) advice on several issues such as, birth preparedness, skilled assisted delivery, essential newborn care, breastfeeding, immunization, and family planning etc. Despite significant differences in coverage of ANC checkup between the two slum areas, one-third of women in both areas were considered to have received a better quality of care (35.3% vs. 31.2%; p <0.001).

**Table 3:** Antenatal Care (ANC) practices by the respondents.

Study Variables		Intervention slum, n = 607		Comparison slum, n = 599		P-value
Received tetanus injection during pregnancy, % (n)*		55.02	(334)	48.08	(288)	0.005
Completed tetanus dose before pregnancy, % (n)*		28.83	(175)	37.40	(224)	
Received iron and folic acid tablet during pregnancy, % (n)*		65.90	(400)	46.91	(281)	<0.001
Period of receiving first ANC, Mean±SD**		3.98	±1.83	5.04	±1.87	<0.001
Received at least one ANC from MTPs ^a^, % (n)*		79.70	(484)	56.80	(340)	<0.001
Received at least one ANC from BRAC CHWs, % (n)*		9.40	(57)	0.80	(5)	<0.001
Received at least four ANCs from MTPs ^a^, % (n) *		42.50	(258)	20.20	(121)	<0.001
Received at least four ANCs from BRAC CHWs, % (n)*		3.00	(18)	0.20	(1)	<0.001
Received at least four ANCs from trained provider, % (n)*^b^		47.60	(289)	21.00	(126)	<0.001
Tests of ANC checkup, % (n)*						
	Pulse rate	76.60	(465)	47.10	(282)	<0.001
	Blood pressure	79.40	(482)	53.90	(323)	<0.001
	Weight measurement	70.20	(426)	50.90	(305)	<0.001
	Height measurement	18.60	(113)	8.70	(52)	<0.001
	Blood test	48.30	(293)	29.90	(179)	<0.001
	Urine test	56.20	(341)	33.90	(203)	<0.001
	Fetus position	77.90	(473)	52.40	(314)	<0.001
	Fundal height	55.00	(334)	26.50	(159)	<0.001
	Fetus heart beat	62.90	(382)	40.90	(245)	<0.001
	Ultrasonography	66.10	(401)	48.20	(289)	<0.001
Received one or more sets of advice ^c^, % (n)*		100.00	(607)	100.00	(599)	---
Quality of care, % (n) *						
	Poor	20.60	(125)	46.20	(277)	<0.001
	Moderate	44.20	(268)	22.50	(135)	
	Better	35.30	(214)	31.20	(187)	

*χ2

** Student’s *t* test

CHW- Community Health Workers; ANC-Antenatal care; MTP- Medically trained providers

^a^ Medically trained providers includes qualified doctor, nurse, FWV, and paramedic

^b^ Trained providers includes BRAC SK in addition to the medically trained providers

^c^Advices include TT injection, dietary intake, resting, iron tablet, folic acid, breastfeeding, newborn care, family planning, not doing heavy work, abstinence, ANC, cleaniness, wearing loose clothes, contact with birth attendant, complications, birth place, buying delivery kit, saving money, keeping clothes for wrapping and wiping, buying misoprostol tablet, preparing transport, keeping phone number of health worker, fixing blood donor, taking to hospital for emergency

Intervention slum: Slums of Narayanganj City Corporation; Comparison slum: Slums of Narsingdi Sadar Municipality

The number of institutional deliveries including caesarean sections and episiotomies were higher in the intervention slums compared to the comparison slums (Table 4). The proportion of MTPs assisted delivery was 53.5% in the intervention slums and 45.4% in the comparison slums (p <0.001). In the case of home deliveries, significantly higher numbers of umbilical cords were cut with sterile blades in the intervention slums compared to their counterpart (91.9% vs. 77.7%; p <0.001) and tied by sterile thread (80.7% vs. 58.7%; p = 0.008). Almost half of the mothers in both slum areas experienced complications during delivery (44.5% vs. 46.6%) and among them more women in the intervention slums sought treatment from MTPs than the comparison slums (76.7% vs. 62.4%; p <0.001). The proportion of respondents who received PNC after 48 hours from MTPs was similar in both intervention and comparison area (44.4% vs. 39.5%; Table 4). However, this proportion improved significantly while the efforts of MTPs and BRAC CHWs combined in the trained providers’ category (47.9% vs. 39.5%; p = 0.005). In the case of home deliveries, very few women from both areas received PNC from MTPs within 48 hours after delivery. However, a significantly higher proportion of women in the intervention slums received PNC from trained providers compared to comparison slums (8.5% vs. 4.7%; p = 0.039).

**Table 4:** Delivery care and Postnatal Care (PNC) practice by the respondents.

Study Variables		Intervention slum, n = 607		Comparison slum, n = 599		P-value
Number of total delivery		576		535		
Mode of delivery, % (n)*						
	Normal	57.30	(330)	67.10	(359)	0.001
	Episiotomy	11.10	(64)	6.00	(32)	
	C-section	31.60	(182)	26.90	(144)	
Birth attendant during last delivery, % (n)*		`	`			
	MTPs ^a^	53.50	(308)	45.40	(243)	0.007
	Trained traditional birth attendant	16.50	(95)	11.00	(59)	<0.001
	Untrained traditional birth attendant	23.10	(133)	37.00	(198)	<0.001
	Urban birth attendant	2.80	(16)	0.00	(0)	<0.001
**Home delivery**						
Home delivery, % (n)*		46.90	(270)	56.30	(301)	<0.001
Attendants at home delivery, % (n)*						
	MTP ^a^	7.80	(21)	3.30	(10)	<0.001
	Trained/ untrained traditional birth attendant	84.10	(227)	85.10	(256)	
	Others ^b^	8.10	(22)	11.60	(35)	
Cutting cord with sterile blade, % (n)*		91.90	(248)	77.7	(234)	<0.001
Tying cord with sterile thread, % (n)*		80.70	(218)	58.5	(176)	0.008
**Delivery complications** ^**c**^						
One or more delivery complications ^c^, % (n)*		44.50	(256)	46.60	(268)	0.465
Seeking treatment from MTPs ^a^ for delivery complications,% (n)*		76.70	(196)	62.40	(167)	<0.001
**PNC within 48 hours after delivery for women**		572		531		
Received PNC from MTPs ^a^, % (n)		44.40	(254)	39.50	(210)	0.103
Received PNC from BRAC CHWs, % (n)*		3.50	(20)	0.00	(0)	0.005
Received at least one PNC from trained provider ^d^, % (n)*		47.90	(274)	39.50	(210)	0.005
**PNC within 48 hours after delivery for home delivery**						
Number of home delivery		270		301		
Received PNC from MTPs ^a^, % (n)*		5.90	(16)	4.30	(13)	0.383
Received PNC from BRAC CHWs, % (n)*		4.10	(11)	0.00	(0)	<0.001
Received at least one PNC from trained provider ^d^, % (n)*		8.50	(23)	4.70	(14)	0.039

*χ2; CHW- Community Health Workers; PNC-Postnatal care; MTP- Medically trained providers

^a^Medically trained providers includes qualified doctor, nurse, FWV and paramedic

^b^Includes self and relatives

^c^Delivery Complications includes any of the symptom such as, substantial bleeding, high fever, high pressure, blurry vision, severe headache, mal position, prolonged labor, retained placenta, ruptured uterus, cord prolapsed, hand/leg prolapsed, cord around neck, convulsion, obstructed labor, mother fainted, perineal tear, still birth

^d^ Trained providers includes BRAC SK in addition to the medically trained providers

Intervention slum: Slums of Narayanganj City Corporation; Comparison slum: Slums of Narsingdi Sadar Municipality

Finally, association between key maternal health service indicators and other explanatory variables of both intervention and comparison slums and all slums together has been presented in Table 5. It found that probability of receiving four or more ANCs from MTPs was positively associated with the wealth of the family of the respondents and this was a dose response relationship. MTP assisted delivery was associated positively with literacy of the mothers (RR 1.15; 95% CI 1.03–1.28); a higher wealth quintile [fourth: (RR 1.28; 95% CI 1.09–1.50) and fifth: (RR 1.33; 95% CI 1.14–1.56)] and four or more ANCs from MTPs (RR 1.44; 95% CI 1.32–1.56). A positive dose response relationship was found between the receipt of PNC within 48-hours by MTPs and the wealth quintiles. Literacy was also associated positively with PNC (RR 1.22; 95% CI 1.03–1.45). Women of higher wealth quintile had 78% higher chance of receiving delivery care from an MTP onset of delivery complication than that of the poorest quintile. This was also associated positively with literacy of the women (RR 1.27; 95% CI 1.07–1.50) and receiving four or more ANC from MTPs (RR 1.69; 95% CI 1.47–1.95). The use of modern family planning methods was associated with exposure to television (RR 1.21; 95% CI 1.006–1.45) and receiving ANC from MTPs (RR 1.15; 95% CI 1.03–1.27).

**Table 5:** Association between indicators and its predictors.

Study Variable		Intervention slum		Comparison slum		All slums	
		a RR	(95% CI)	a RR	(95% CI)	a RR	(95% CI)
**Four and more ANC by MTPs** ^a^							
Can read and write		1.26	1.30–2.27*	1.26	0.80–1.98	1.54	1.21–1.94
Husband can read and write		1.21	0.94–1.54	1.83	1.10–3.03*	1.32	1.04–1.66*
Age of first conception		0.99	0.61–1.61	0.88	0.42–1.80	0.92	0.60–1.41
Wealth index							
	2^nd^ quintile	1.32	0.83–2.10	4.70	1.66–13.29*	1.81	1.19–2.77*
	3^rd^ quintile	1.87	1.24–2.83*	3.37	1.14–9.91*	2.18	1.45–3.27*
	4^th^ quintile	1.96	1.30–2.96*	5.67	2.02–15.93*	2.52	1.69–3.76*
	5^th^ quintile	2.25	1.50–3.37*	7.61	2.73–21.20*	3.12	2.11–4.63*
Watch TV		1.03	0.71–1.49	1.17	0.62–2.21	1.16	0.82–1.65
NGO involvement		0.94	0.77–1.15	0.98	0.69–1.40	0.93	0.77–1.12
**Birth assisted by MTPs** ^a^ **/SBAs** ^b^							
Can read and write		1.11	1.0002–1.23*	1.18	0.97–1.44	1.15	1.03–1.28*
Husband can read and write		---	---	1.21	0.98–1.48	1.04	0.94–1.14
Age of first conception		0.86	0.62–1.18	---	---	0.98	0.81–1.19
Wealth index							
	2^nd^ quintile	1.12	0.92–1.35	1.10	0.81–1.50	1.09	0.92–1.30
	3^rd^ quintile	1.18	0.99–1.41	1.05	0.76–1.46	1.17	0.99–1.38
	4^th^ quintile	1.19	1.04–1.42*	1.45	1.09–1.94*	1.28	1.09–1.50*
	5^th^ quintile	1.25	1.06–1.48*	1.51	1.13–2.02*	1.33	1.14–1.56*
Read newspapers		0.97	0.93–1.01	1.02	0.88–1.19	0.98	0.91–1.00
Listen to Radio		---	---	1.20	1.05–1.36*	---	---
Watch TV		---	---	0.93	0.74–1.18	---	---
NGO involvement		---	---	0.99	0.85–1.14	1.00	0.94–1.06
One or more child deaths		1.05	1.02–1.09*	---	---	1.04	0.98–1.11
Four and more ANC by MTPs ^a^		1.49	1.33–1.67*	1.23	1.08–1.41*	1.44	1.32–1.56*
**PNC within 48 hours by MTPs** ^a^							
Can read and write		1.20	0.98–1.45	1.45	1.09–1.93*	1.22	1.03–1.45*
Husband can read and write		1.20	0.98–1.45	1.02	0.79–1.33	1.11	0.94–1.30
Age of first conception		0.89	0.57–1.39	1.40	1.02–1.93*	1.10	0.87–1.39
Wealth index							
	2^nd^ quintile	1.27	0.90–1.77	1.78	1.13–2.81*	1.40	1.06–1.84*
	3^rd^ quintile	1.36	0.99–1.86	1.78	1.11–2.84*	1.52	1.16–1.99*
	4^th^ quintile	1.49	1.10–2.03*	2.18	1.39–3.42*	1.72	1.32–2.23*
	5^th^ quintile	1.82	1.35–2.44*	2.46	1.56–3.87*	1.97	1.52–2.55*
Read newspapers		---	---	---	---	1.33	1.17–1.57*
Watch TV		1.43	0.97–2.11	1.39	0.91–2.13	1.36	1.03–1.80*
NGO involvement		0.82	0.68–0.98*	0.95	0.76–1.19	---	---
One or more child deaths		1.05	0.77–1.44	1.23	0.90–1.70	---	---
**Treatment seeking from MTPs** ^a^ **for delivery complications**							
Can read and write		1.35	1.05–1.73*	1.17	0.93–1.46	1.27	1.07–1.50*
Age of first conception		1.08	0.91–1.30	1.09	0.91–1.31	1.09	0.95–1.24
Wealth index							
	2^nd^ quintile	1.26	0.81–1.95	1.64	1.03–2.63*	1.44	1.05–1.98*
	3^rd^ quintile	1.65	1.11–2.46*	1.84	1.15–2.94*	1.79	1.30–2.38*
	4^th^ quintile	1.66	1.11–2.47*	2.06	1.31–3.25*	1.85	1.37–2.49*
	5^th^ quintile	1.50	1.11–2.47*	2.08	1.31–3.29*	1.78	1.31–2.40*
Watch TV		1.10	0.76–1.58	1.23	0.82–1.85	1.14	0.87–1.50
Four and more ANC by MTPs ^a^		1.77	1.41–2.21*	1.79	1.48–2.16*	1.69	1.47–1.95*
**Use of modern family planning methods**							
Can read and write		1.09	0.95–1.26	0.99	0.82–1.19	1.04	0.93–1.16
Age of first conception		0.92	0.66–1.28	0.96	0.69–1.33	0.94	0.74–1.18
Wealth index							
	2^nd^ quintile	0.80	0.66–0.99*	0.92	0.71–1.18	0.83	0.71–0.98*
	3^rd^ quintile	0.88	0.73–1.06	0.90	0.69–1.18	0.89	0.76–1.04
	4^th^ quintile	0.89	0.74–1.07	0.97	0.75–1.26	0.90	0.77–1.05
	5^th^ quintile	0.82	0.68–1.00	0.92	0.70–1.22	0.84	0.71–0.99*
Watch TV		1.01	0.82–1.25	1.44	1.05–1.97*	1.21	1.006–1.45*
NGO involvement		1.07	0.94–1.23	1.16	0.98–1.37	1.10	0.99–1.22
Four and more ANC by MTPs ^a^		1.07	0.94–1.21	1.04	0.85–1.27	1.15	1.03–1.27*

RR = Risk Ratio;a RR = Adjusted Risk Ratio; CI–Confidence Interval

*P<0.05, was calculated by using log bi-nominal model

^a^ Medically trained providers includes qualified doctor, nurse, FWV and paramedic

^b^ Skilled birth attendant includes qualified doctor, nurse, FWV and paramedic

Intervention slum: Slums of Narayanganj City Corporation; Comparison slum: Slums of Narsingdi Sadar Municipality

## Discussion

Findings of this study revealed a significant difference in the maternal health service indicators between intervention and comparison slums. We aimed to capture the differences of the maternal health service indicators between the intervention areas where the MANOSHI programme was in its initial phase and a non-intervention area. To the best of our knowledge, this study is the first of its kind, which measures the maternal health service indicators in the slums of municipality in Bangladesh. Social determinants that were likely to affect access to maternal health services, such as poverty and literacy, were similar in both the intervention and comparison slums. Importantly, the determinants of using health care services were similar to those previously reported in other disadvantaged groups in Bangladesh [29] and in many other developing countries in Asia and Africa [30–35].

All the maternal health care practices namely four or more ANC from the MTPs, institutional delivery, skilled assisted delivery, and PNC from an MTP, within 48 hours after delivery, in the intervention slums were lower than the MANOSHI endline survey [21] and better than the findings of slum in a recent urban health survey [14]. In the Narsingdi Sadar Municipality, the proportion of women who received these services was similar to other municipality areas as shown in the recent survey [14] except the four or more ANC. Since this survey was conducted in slum areas it did not represent the whole population because of poor knowledge of services and poverty, people may be reluctant to access these services.

Although the contraceptive prevalence rate in the intervention area was higher, adolescent motherhood was prevalent in both areas. In Southeast Asia and Sub-Saharan Africa family planning programme has been effective for the last four decades [36]. Consequence of pregnancy at a young age can be inadequate weight gain during pregnancy, and poor maternal and child outcome [37–40]. The results of this study were also in agreement with an earlier report associating a higher proportion of neonatal death with teenage pregnancies [37].This finding indicated the urgency of sensitising adolescent married girls living in slums to prevent early pregnancy. Moreover, the contraceptive prevalence rate was associated with exposure to mass media, suggesting that promotion of permanent family planning methods by the CHWs would bring success in birth spacing [9, 41].

ANC checkup consists of several examinations, including physical, biochemical, and ultrasound. These are required for screening of high-risk pregnancies and obtaining the best possible pregnancy outcomes. In addition, earlier studies showed that counseling among mothers during ANC checkups on birth preparedness, skilled assisted delivery, essential newborn care, and child nutrition was effective for preventing morbidity of both mothers and newborns [42–47]. All together determined the quality of ANC checkups, which showed there was room for improving quality of care in the intervention area. However, more ultrasound examinations in intervention slums during pregnancy did not enhance the quality of care rather it reflected that they had more exposure with private facilities, and diagnostic centres, which increased the number of C-sections among them significantly [48].

Still, slum communities in Bangladesh tend to use apparently cheap, easily accessible informal sectors for seeking and obtaining health-care services, which was true for the current study [16]. Cultural beliefs play important roles in reinforcing the use of home deliveries without skilled assistance [16, 49]. Since these factors may increase the risk of maternal mortality and post-partum morbidity [50], it is extremely important that women should be made aware of the importance of utilising skilled attendance during delivery. Although the community skilled birth attendant programme has been in existence for over a decade, it has only shown limited efficacy within the community [10]. The BRAC MANOSHI midwives are working now. Therefore, increasing the number of deliveries at the BRAC delivery facilities would ensure safe delivery. However, they are only able to conduct normal deliveries and have been told to refer delivery cases with complications to the facility with EmOC where BRAC has a linkage. However, unpleasant experiences in the public facilities increases the stigma around institutional delivery among poor slum dwellers (15, 16). As a result, they prefer home delivery that is assisted by unskilled providers. In order to reduce both maternal and neonatal mortality women with risky pregnancy outcomes must use the services of EmOC. Therefore, the referral system between BRAC MANOSHI programme and facilities with EmOC need to be strengthened.

As found in previous studies in Bangladesh, inequality was observed in the assistance provided by skilled birth attendants during delivery between the poorest and the richest in the intervention slums [29, 51]. It demonstrated an urgent requirement to encourage the poorest women in the intervention area to give birth at BRAC delivery facilities. Nevertheless, seeking treatment for delivery complications did not vary across the socio-economic range. They were, however, independently associated with maternal literacy and women receiving four and more ANC from the MTPs. An earlier Bangladeshi study reported that families were less likely to use MTPs for conducting delivery unless complications had already occurred [52]. However, whilst the MANOSHI program was previously effective in reducing first and second delays, this was not the case for third delays in accessing EmOC [12]. These results illustrate that community awareness and a strong referral linkage between MANOSHI programme and EmOC for all complications is important to reduce preventable deaths in the intervention area.

The significance of receiving PNC within 48-hours of birth in reducing maternal and neonatal morbidity is well established [49, 53, 54]. In the case of home births, negligible numbers of women received comparable PNC care thus indicating an urgent requirement for improved access to PNC. As found in earlier studies, this study also found inequity in receiving PNC services between high and low wealth quintiles and illiteracy [29, 55]. However, in the earlier study distance of health care facilities hindered using PNC services [56], which was not a fact for the Narayanganj City Corporation as health facilities were available around the slums, rather financial and cultural barriers of this community made it difficult to provide PNC facilities outside the home during the early postnatal period. A recent study found that it was possible to improve PNC services in rural Bangladesh through a door-to-door service provided by the BRAC CHWs [29]. Furthermore, CHWs have also been shown to be valuable within the rural community, in Bangladesh and India, for conducting deliveries, identifying postpartum complications, and arranging referrals [49, 54, 57, 58].

Although increasing use of MTPs is helping to improve maternal and child care, the current study still identified a serious gap in the use of their services in both intervention and comparison areas. In Bangladesh, BRAC CHWs have increased rapidly to help improve access to basic community health services [59]. Since, slum dwellers are increasing in the urban areas it is urgent to improve utilisation of BRAC CHWs through the MANOSHI programme. The programme must contribute to the promotion of modern family planning methods, improving skilled assisted delivery, ANC and PNC checkups for women and their neonates [20]. Although CHWs are doing tremendous work to improve maternal and neonatal health in the MANOSHI intervention area, they were not satisfied with their salary [60]. This could be a potential cause of the frequent drop out of skilled CHWs in the intervention area, which could be one of the challenges for the MANOSHI programme. In order to address the challenge the management of the MANOSHI programme need to consider the financial incentive and an improved social status for CHWs [60].

Data on age of the respondents, age at first marriage, age at first conception and maternal health-services that were received during the last pregnancy was collected using the recall method. Therefore, overestimation, underestimation, and misclassification might persist due to information and recall bias. To reduce recall bias, we asked to see records of some information, for example, birth certificate, certificate of secondary school certificate examination, ANC card, doctor’s prescription, and test reports. Moreover, we used a validated questionnaire. For reduction of inter and intra observer variation, data collectors received a comprehensive training and a supervisory team always monitored their activities. Since the MANOSHI programme was implemented in all city corporations in Bangladesh, it was not possible to select slums of another city corporation as comparison area. Comparison slums were matched with intervention slums according to the poverty scale and randomisation performed during the selection of samples to reduce confounders. As this was a cross-sectional survey, temporal relationships cannot be explained. Therefore, results of this study should be interpreted with caution. Another limitation was not randomly assigning the intervention and comparison group. However, participants were randomly selected from the study areas based on the pre-determined criterion in order to minimize the selection bias. The strength of this study includes blinding both the interviewers and supervisors to the rationale of the study to avoid differential misclassification of outcome. We recruited female interviewers with a view to minimise the gender-biased response. Although we enrolled women from two different slum areas, as a proxy for socioeconomic status they were not significantly different (p = 0.488) in different quintiles of wealth index between the intervention and comparison slum areas.

## Conclusions

The MANOSHI programme intervention area in Narayangonj City Corporation had higher ANC coverage compared to comparison area. Still half of the deliveries in the intervention area took place at home with the assistance of unskilled attendants and of which very few received PNC-checkup within recommended period. The door-to-door service of the MANOSHI programme through CHWs targeting urban slum women needs to be scaled up for the poor and illiterate in order to ensure safe motherhood.
